# Pterional vs. lateral supraorbital approach in the management of middle cerebral artery aneurysms: insights from a phantom model study

**DOI:** 10.1007/s10143-024-02518-6

**Published:** 2024-07-22

**Authors:** Amir Amini, Vanessa M. Swiatek, Klaus-Peter Stein, Ali Rashidi, I. Erol Sandalcioglu, Belal Neyazi

**Affiliations:** Department of Neurosurgery, University Hospital Magdeburg, Otto-von-Guericke University, Leipziger Str. 44, 39120 Magdeburg, Saxony Anhalt, Germany

## Abstract

The pterional approach has traditionally been employed for managing middle cerebral artery (MCA) aneurysms. With potential benefits like reduced surgical morbidity and improved postoperative recovery, the lateral supraorbital approach (LSO) should be considered individually based on aneurysm morphology, location and patient-specific variations of the MCA anatomy, which requires considerable technical expertise traditionally acquired through years of experience. The goal of this study was the development and evaluation of a novel phantom simulator in the context of clinical decision-making in the managmement of MCA aneurysms. For this purpose, high-fidelity simulators inclusive of MCA models with identical M1- and bifurcation aneurysms were manufactured employing 3D reconstruction techniques, additive manufacturing and rheological testings. Medical students, neurosurgical residents, and seasoned neurosurgeons (*n* = 22) tested and evaluated both approaches. Participants’ performances and progress over time were assessed based on objective metrics. The simulator received positive ratings in face and content validity, with mean scores of 4.9 out of 5, respectively. Objective evaluation demonstrated the model’s efficacy as a practical training tool, particularly among inexperienced participants. While requiring more technical expertise, results of the comparative analysis suggest that the LSO approach can improve clipping precision and outcome particularly in patients with shorter than average M1-segments. In conclusion, the employed methodology allowed a direct comparison of the pterional and LSO approaches, revealing comparable success rates via the LSO approach while reducing operation time and complication rate. Future research should aim to establish simulators in the context of clinical decision making.

## Introduction

The pterional approach [1] has been the standard technique employed for the management of middle cerebral artery (MCA) aneurysms for decades, providing wide exposure of the anterior and middle cranial fossae, sellar and parasellar regions, superior orbital fissure, and cavernous sinus [2–4]. However, the risk of temporalis muscle atrophy, damage to the frontal branch of the facial nerve, and cosmetic issues has limited this extremely versatile approach [5].

Modifications of the standard pterional approach have become popularized over the last 20 years, including mini-pterional [6, 7] and lateral supraorbital (LSO) craniotomies [8, 9]. The LSO offers several potential benefits over the pterional approach for the surgical treatment of unruptured MCA-bifurcation aneurysms. First, it offers comparable surgical exposure despite a smaller skin incision and craniotomy. This may result in less surgical morbidity and improved postoperative recovery, which is a significant advantage in patient-centered care. Second, the improved ergonomics offered by the LSO approach may reduce surgical time and improve the precision of aneurysm clipping.

The choice of surgical approach should be tailored to the individual patient and the specific characteristics of the aneurysm. Each case requires specific microsurgical skills and techniques for a safe and effective dissection. Mastering this crucial technique requires considerable expertise and technical skills that were traditionally acquired through years of experience and rigorous training. As traditional teaching methods [10, 11] cannot provide adequate opportunities for deliberate practice and skill development, alternative training opportunities are needed to accelerate the surgical learning curve [12–14].

Simulators can provide a safe, controlled, and risk-free environment for training, skill development, and mastery of intricate surgical techniques [15]. Moreover, if implemented correctly, high quality phantom simulators have the potential to explore new techniques and approaches in a safe environment, allowing a shift towards a more pathology and patient-specific surgical treatment. However, the effectiveness of simulation training heavily relies on the quality and realism of the simulation techniques employed.

This study aims to develop and evaluate a high-fidelity simulator for the microsurgical management of MCA aneurysms in the context of clinical decision making. For this purpose, meticulously reconstructed models of the skull, brain, and meninges inclusive of MCA models with identical M1- and MCA-bifurcation aneurysms were manufactured employing 3D reconstruction techniques, additive manufacturing and rheological measurements. The simulator facilitates an unprecedented direct comparative analysis of two distinct surgical approaches in the management of identical MCA aneurysms, a comparison previously unachievable due to the unique nature of each clinical scenario. To meet the requirements for an effective training and decision-making tool, the simulator was assessed by medical students, neurosurgical residents, and experienced neurosurgeons based on subjective and objective evaluation criteria.

## Materials and methods

This study protocol adheres to the SQUIRE 2.0 guidelines.

### Ethics approval

The study protocol was conducted in compliance with the Declaration of Helsinki and approved by the ethics committee of the Otto-von-Guericke University Magdeburg (Ethics vote number: RENOVA 94/20).

### Statement of human and animal rights

This article does not contain any studies with human or animal subjects.

### Statement of informed consent

Written informed consent was obtained from the patient for their anonymized information to be published in this article.

### Data acquisition

After obtaining the approval of the local ethics committee, imaging datasets (computed tomography angiography [CTA], and magnetic resonance imaging [MRI]) of a 52-year-old male with an incidental aneurysm of the left middle cerebral artery (MCA) were used for thresholding-based segmentation and reconstruction of the skull, brain and the circulus arteriosus willisi (CAW).

### Construction of the phantom

The creation of the phantom combined digital reconstructions and post-processing with additive manufacturing, material research and handcrafting.

**Fig. 1:**
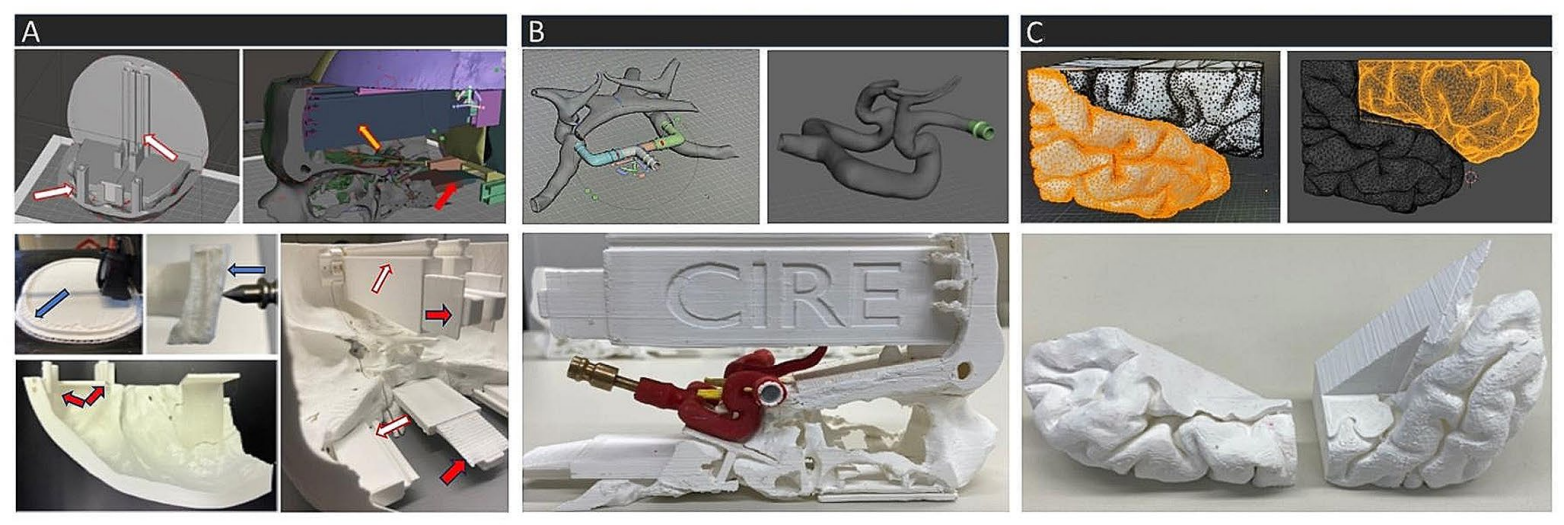
(**A**) digital reconstruction, postprocessing and additive manufacturing of the skull with modifications to the filler density of the model to simulate the tabula externa and interna (blue arrows). Plug-in (red arrows) and slide mechanism (white arrows) incorporated in the skull allowing swift de- and reattachment of the replaceable parts (**B**) reconstruction and digital modelling of the CAW in *MeshMixer* and *Blender* designed to remain in the phantom’s central console (**C**) meticulous segmentation and reconstruction of the SF in frontal and temporal lobe parts for anatomical accuracy and to facilitate the casting process

The skull was reconstructed from the patient’s CT-angiography data by greyscale boundaries using the freely available 3D reoncstruction software *InVesalius3* (Centro de Tecnologia da Informação Renato Archer (CTI)) and subsequently refined with the open-source graphics software tools *MeshMixer* (Autodesk, Inc.), and *Blender* (Blender Foundation - Nonprofit organization) to reduce production costs and time expenditure by incorporating reusable and detachable parts via slide and plug-in mechanisms (Fig. 1A).

The CAW was segmented from the patient’s contrast-enhanced T1 weighted MRI dataset on the free cross-plattform application *MeVisLab* (MeVis Medical Solutions AG, Bremen, Germany) employing threshold-based techniques [16]. Subsequent mesh smoothing and adjustments were permormed with *MeshMixer* and *Sculptris 1.02* (Pixologic, Inc., www.sculpteo.com). The CAW was designed to remain in the centrale console of the skull with magnetic connectors integrated into the proximal M1-segments to facilitate a swift attachment and detachment of the aneurysm models (Fig. 1B).

The reconstruction of the brain from the patient’s MRI datatset was executed with the open-source software *Freesurfer* (Harvard University, Cambridge, Massachusetts, USA). Additional manual segmentation was required to accurately replicate the lateral sulcus and generate an anatomically precise negative mold and facilitate the subsequent casting of the Sylvian fissure (SF) models. To achieve this, the SF mesh was manually divided into two segments in Blender, determined by hand-selected points demarcating the pial surfaces of the temporal and frontal lobes within the sulcus (Fig. 1C).

Additive manufacturing of the skull, CAW and SF models were executed on a desktop 3D printer (*Raise3D Pro2 dual extrusion* by Raise 3D Technologies, Inc.) using standard 1.75 mm PLA filaments. The rigidity disparities between compact and cancellous bone druing the drilling process were ensured by adjusting the infill density of the skull at 15%.

Mimicking the tactile properties of the living brain was a challenging process based on previous studies [17] and involving a substudy with subjective and objective material research and evaluations. Candle gel, identified by six experienced neurosurgeons for its similar tactile properties to the brain tissue [18, 19] as encountered during surgery, circumvents the limitations associated with previous gelatin-based models while offering a more sustainable and ethically sound option.

**Fig. 2:**
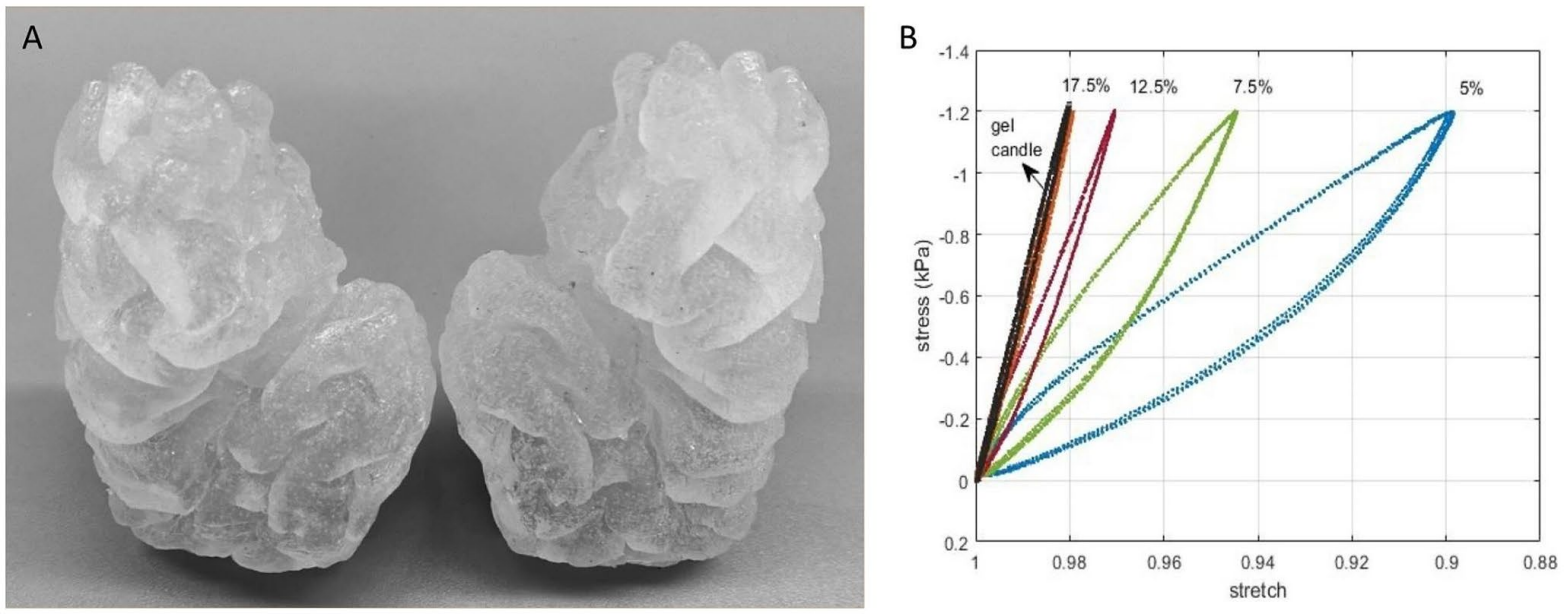
(**A**) anatomically and tactilly accurate replication of the Sylvian fissure. The candel gel-based model is durable, portable, and reusable for weeks without losing its haptic properties (**B**) graph of stretch vs. stress derived by implementing compression stress of (a) 0.2 kPa, (b) 0.6 kPa, and (c) 1.2 kPa on candle gel samples with comparable rheological results as previously as life-like established findings involving 260 Bloom gelatin samples in 17.5% concentrations

The result is an anatomically and tactilly accurate, reusable replication of the SF (Fig. 2A). The subjective evaluation was further validated through rheological assessments (Fig. 2B).

**Fig. 3:**
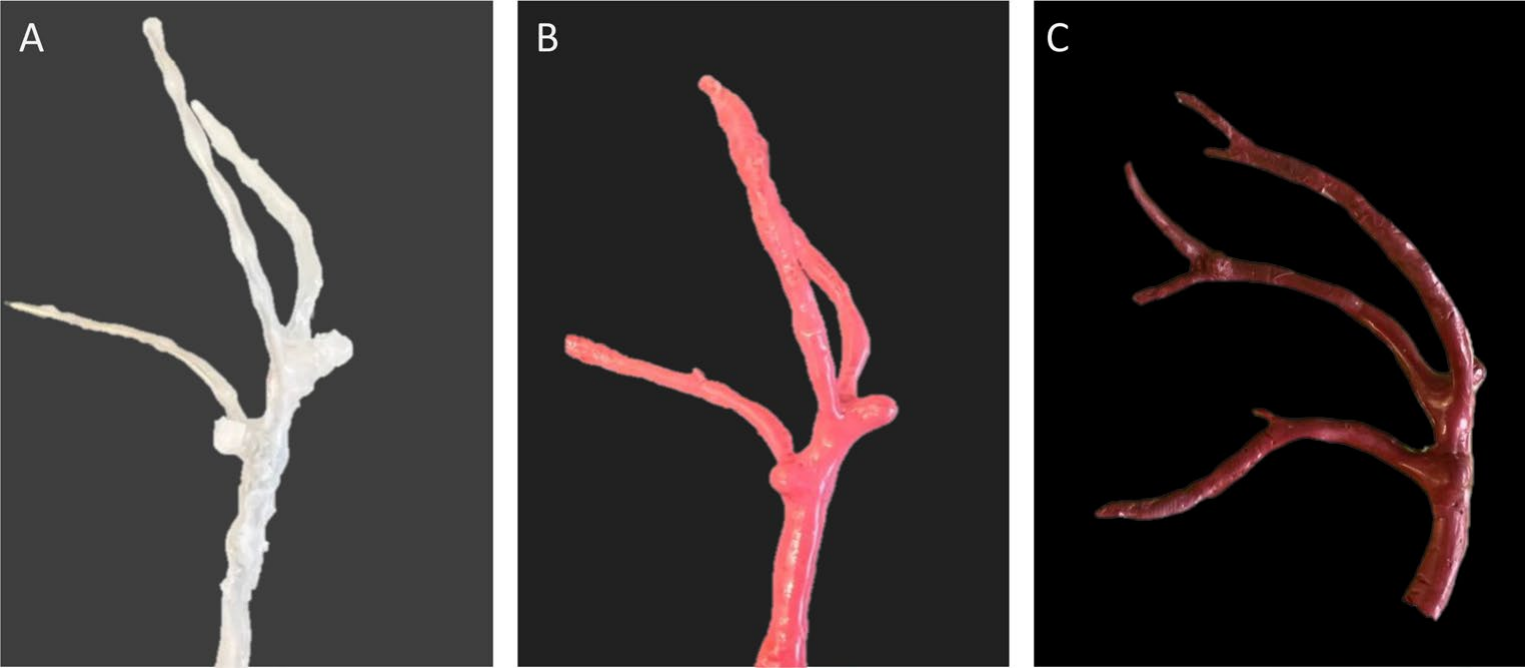
Modeling process of the middle cerebral arteries with bifurcation aneurysms located at 8 mm and 14 mm representing short and average M1 length **(A)** paraffin wax model **(B)** coating of the model with thin layers of liquid latex **(C)** finished left-sided MCA-model with aneurysms

MCA models with identical aneurysms located at M1 and MCA-bifurcation were handcrafted using paraffin wax and coated with two thin layers of liquid latex. After a detaliled shaping process, the models were then bathed in water with temperatures between 65–70 °C to wash out the paraffin wax (Fig. 3A–C).

**Fig. 4:**
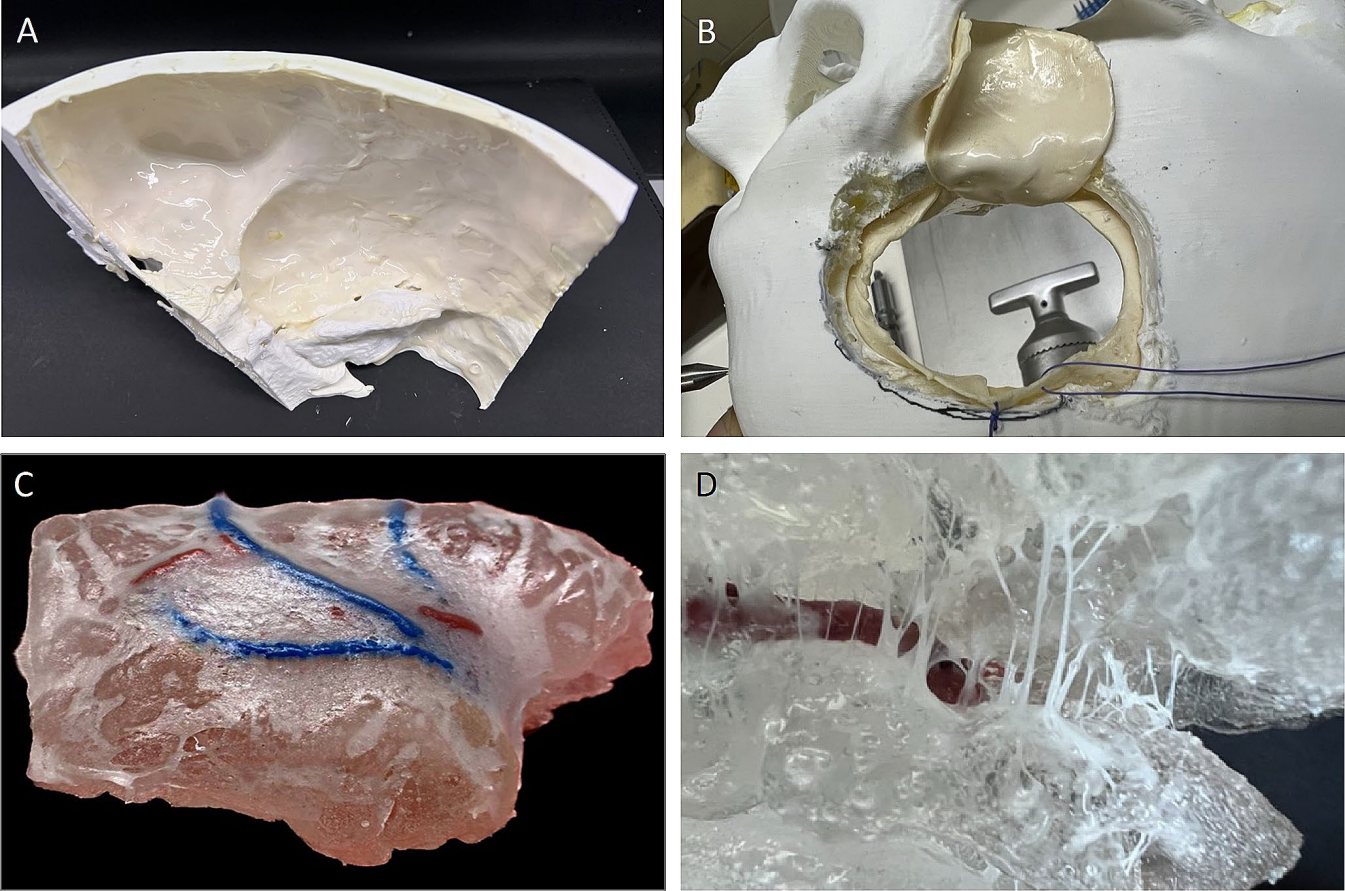
**(A)** application of latex-based dura to the lateral skull base and **(B)** simulation of craniotomy and dural opening **(C)** finished arachnoid membrane applied to the SF with **(D)** anatomically accurate incorporation of arteries and aneurysms within the lateral sulcus

The simulation of the meninges involved applying a latex layer to mimic the dura mater, enhanced with a coalescing agent for better adhesion (Fig. 4A). The web-like texture of the arachnoid mater was recreated using a blend of synthetic resin adhesive, latex, and glycerin, meticulously applied to the SF to achieve a natural, wet look (Fig. 4B).

### Simulator assembly

**Fig. 5:**
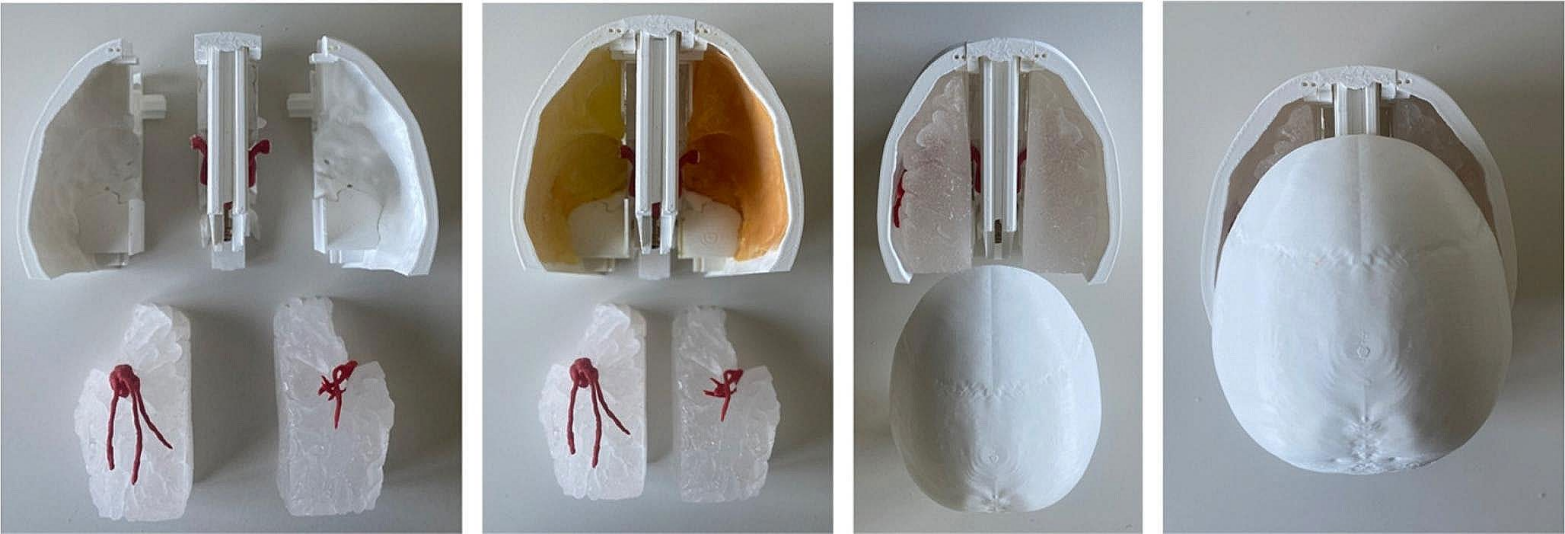
Model assembly with giant MCA aneurysm models (not included in this study)

The initial step of the assembly involves attaching the dura mater to the interchangeable lateral skull base models. Concurrently, the middle cerebral artery aneurysm models, and optionally cerebral veins, are carefully placed within the SF models. After applying the arachnoid membrane, the SF models are positioned in the lateral skull bases which are then connected to the central console via clip mechanisms. During this process, the aneurysm model establishes a magnetic connection with the CAW model placed on the central console. In the final assembly stage, the skull base and central console are securely attached to the rest of the phantom via integrated rail-slide systems. The assembly process is depicted in Fig. 5.

### Study design

Three groups of participants (*n* = 22) with varying levels of neurosurgical experience were recruited for this study (Table 1).

- Novice group (*n* = 12): 4th and 5th -year medical students (MS).
- Advanced group (*n* = 6): 4th and 5th -year neurosurgical residents (NR).
- Expert group (*n* = 4): neurosurgeons (NS) specialized in vascular neurosurgery.

**Table 1:**
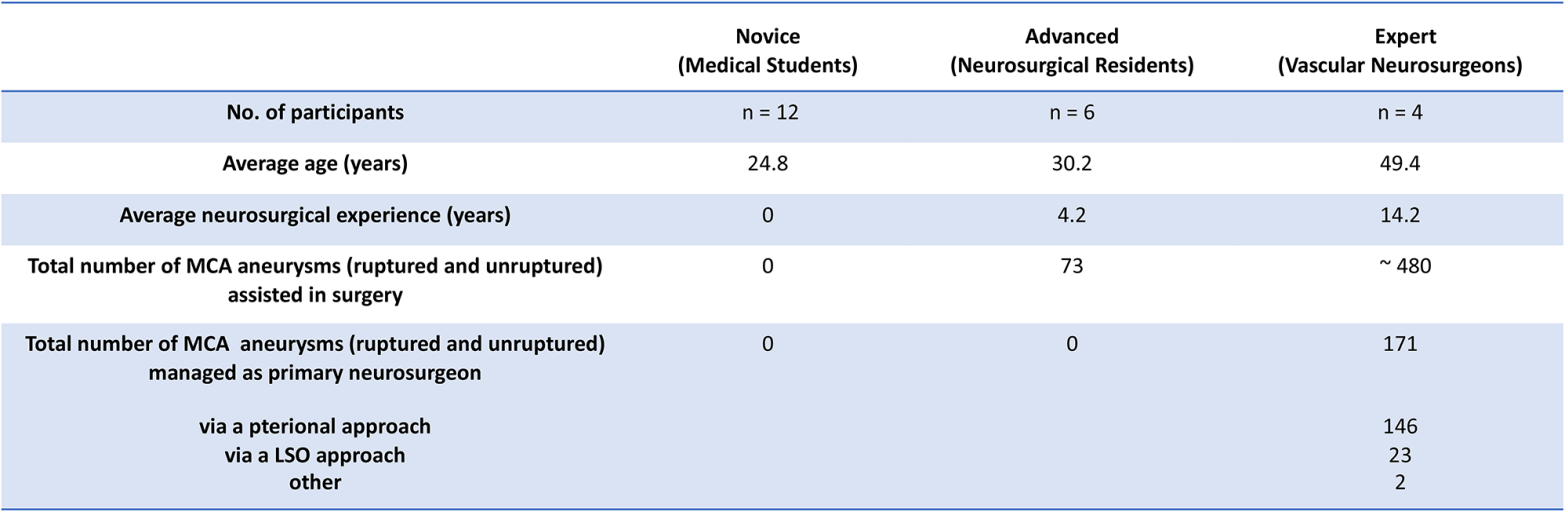
Average age, experience in neurosurgery and microneurosurgical management of MCA aneurysms of participants (*n* = 22)

### Simulation setting

**Fig. 6:**
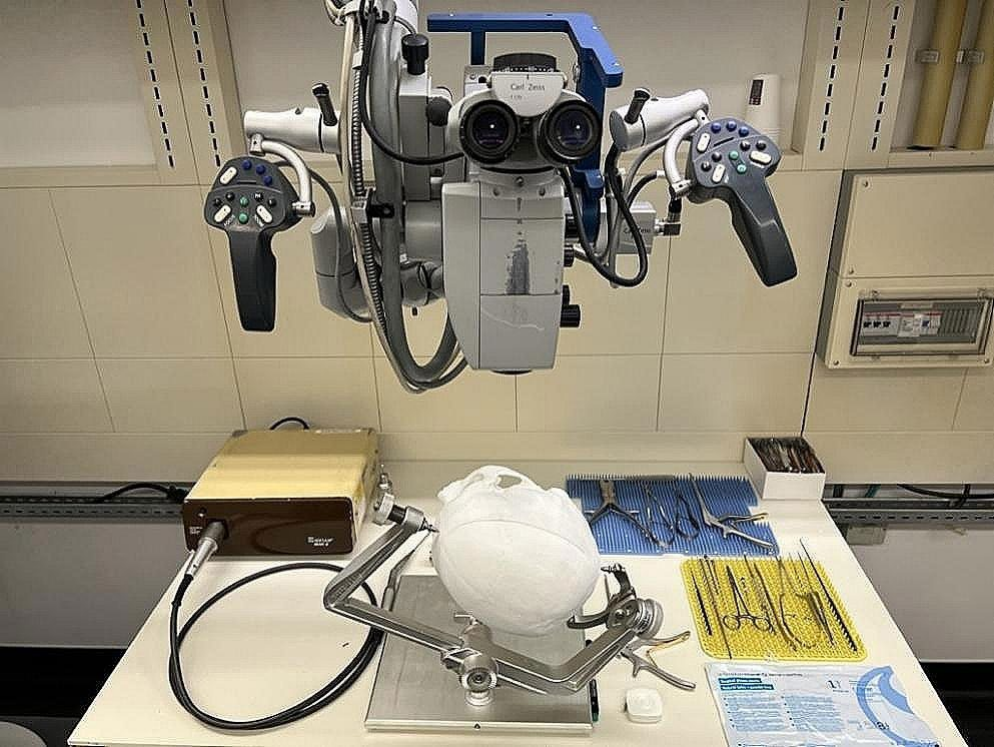
Simulation setup

The simulation took place at the microneurosurgical laboratories of the department of neurosurgery, where a training environment with a full-functioning operating theatre is provided. Simulations were executed using a ZEISS OPMI Neuro NC-4 (Fig. 6). A full set of neurosurgical instruments including drills, scalpels, forceps, scissors, bone punches, aneurysm clips, and clip appliers were organized on a surgical tray within a hand’s reach.

**Fig. 7:**
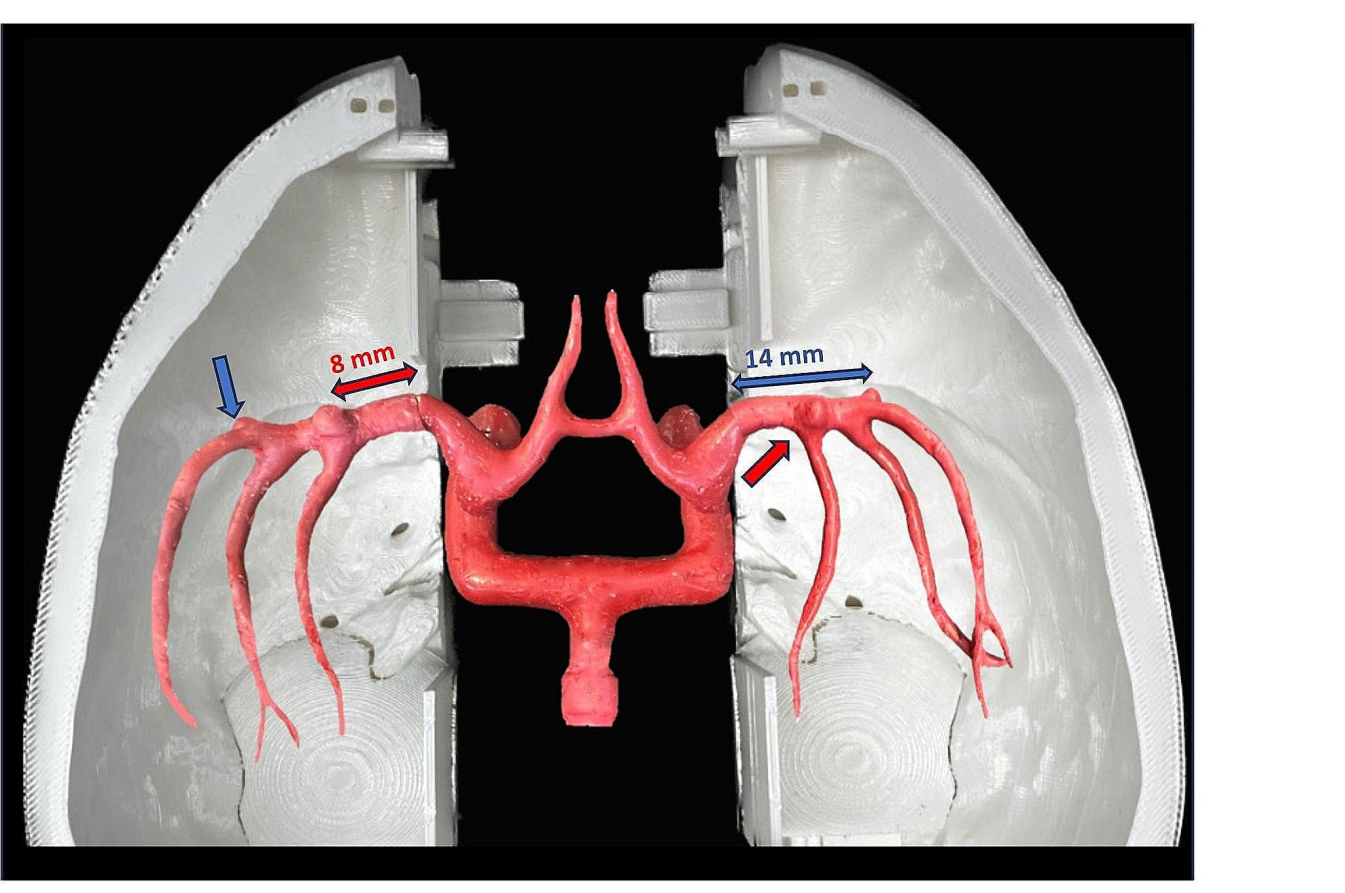
Phantom model with identical MCA-aneurysm models and locations on both sides facilitating the comparative evaluation of the pterional and LSO approaches

To allow a direct comparison between the two approaches, identical MCA models fitted with two aneurysms placed in the M1- and MCA-bifurcation segments, were used in this study. The length of the M1-segment was set at 14 mm [20] where the MCA-bifurcation aneurysm was placed. A second aneurysm was placed at 8 mm length, representing an MCA-bifurcation aneurysm with a shorter M1-segment (Fig. 7).

### Simulation process

**Fig. 8:**
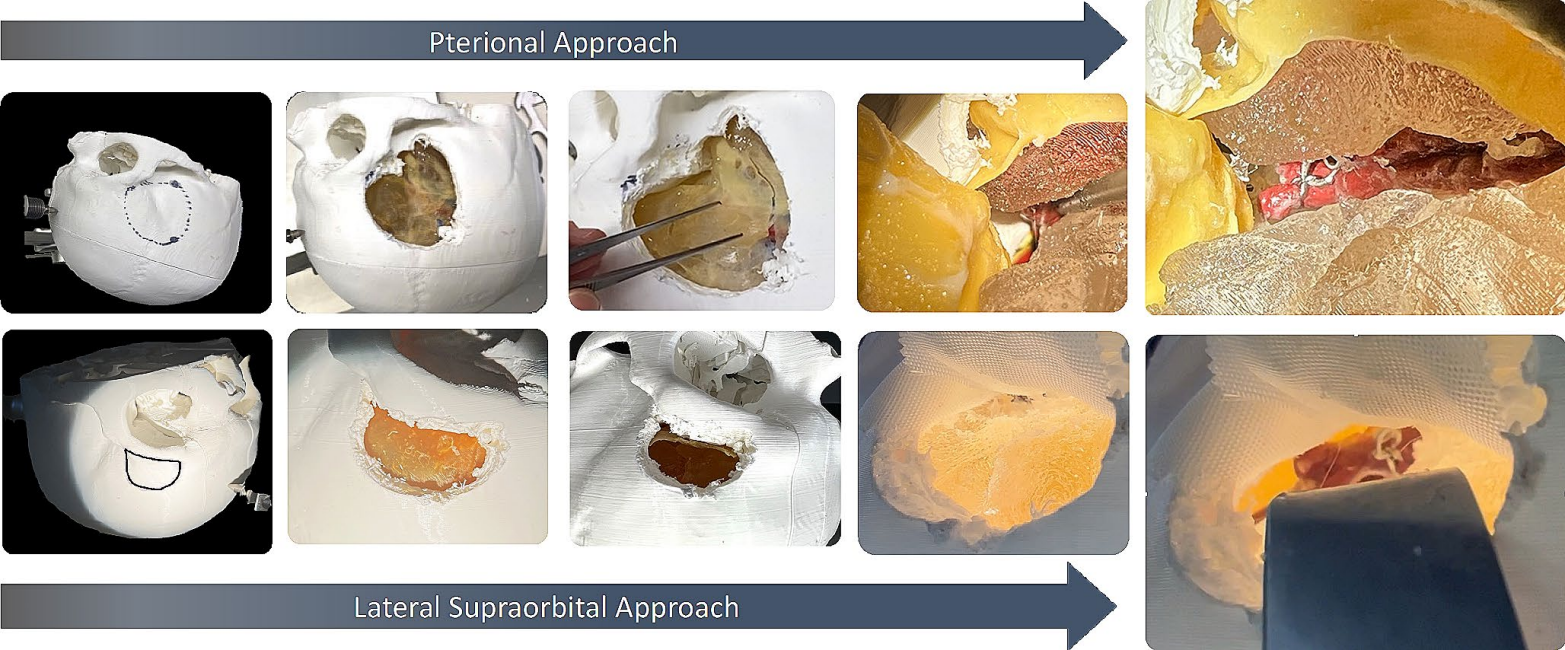
Simulation process starting witg the positioning of the head, craniotomy planning, burr hole and craniotomy placements, dural incision and opening, microsurgical dissection of the SF, visualization of key anatomical landmarks, target exposure, proximal control and clipping of aneurysms

The simulation was preceded by an introduction explaining the principles and key steps of MCA aneurysm clipping. The surgical approach was predefined to the standard pterional and the lateral supraorbital approaches. All medical students received additional instructions on the operating microscope and microsurgical instruments. The participants started the simulation with the positioning of the head in a 3-pin immobilization device. Craniotomy and dural incision were performed. After visualizing the aneurysm, a clip was chosen and placed on the neck of the aneurysm. Each participant performed the procedure twice, with either approach on one side of the phantom. Both approaches were repeated after a period of three to five days. Per attempt, each participant was given one chance to clip each aneurysm with either one or two clips of choice (Fig. 8).

### Simulation assessment

The simulations were directly followed by a questionnaire for the assessment of face and content validity derived on 5-point Likert scales. All participants (*n* = 22) were asked to gauge their attitude towards the simulator. Neurosurgical residents and neurosurgeons rated the simulators’ usefulness in developing technical skills. Experienced neurosurgeons rated the simulators’ realism and accuracy.

The expert group further compared both approaches regarding the surgical exposure of key anatomical structures, accessibility of MCA-bifurcation aneurysms, the ease of surgical manipulation, and overall surgical experience.

All participants were observed during the simulations and assessed by two independent neurosurgeons based on the Objective Structured Assessment of Aneurysm Clipping Skills (OSAACS) [21] (Table 2). OSAACS rates surgical clipping skills based on user performance during simulation to evaluate progress in training and differentiate between novice and advanced surgeons (construct validity). The tool was modified to include specific assessment criteria for correct head positioning and craniotomy placement.

Potential intraoperative complications were recorded independently from the objective assessment of participants’ technical skills, and categorized in three main steps of the procedure:

- Craniotomy: fragmented bone flap?
- Dural opening/Craniotomy: dural tear?
- Clipping: unintentional occlusion of vessel?

**Table 2:**
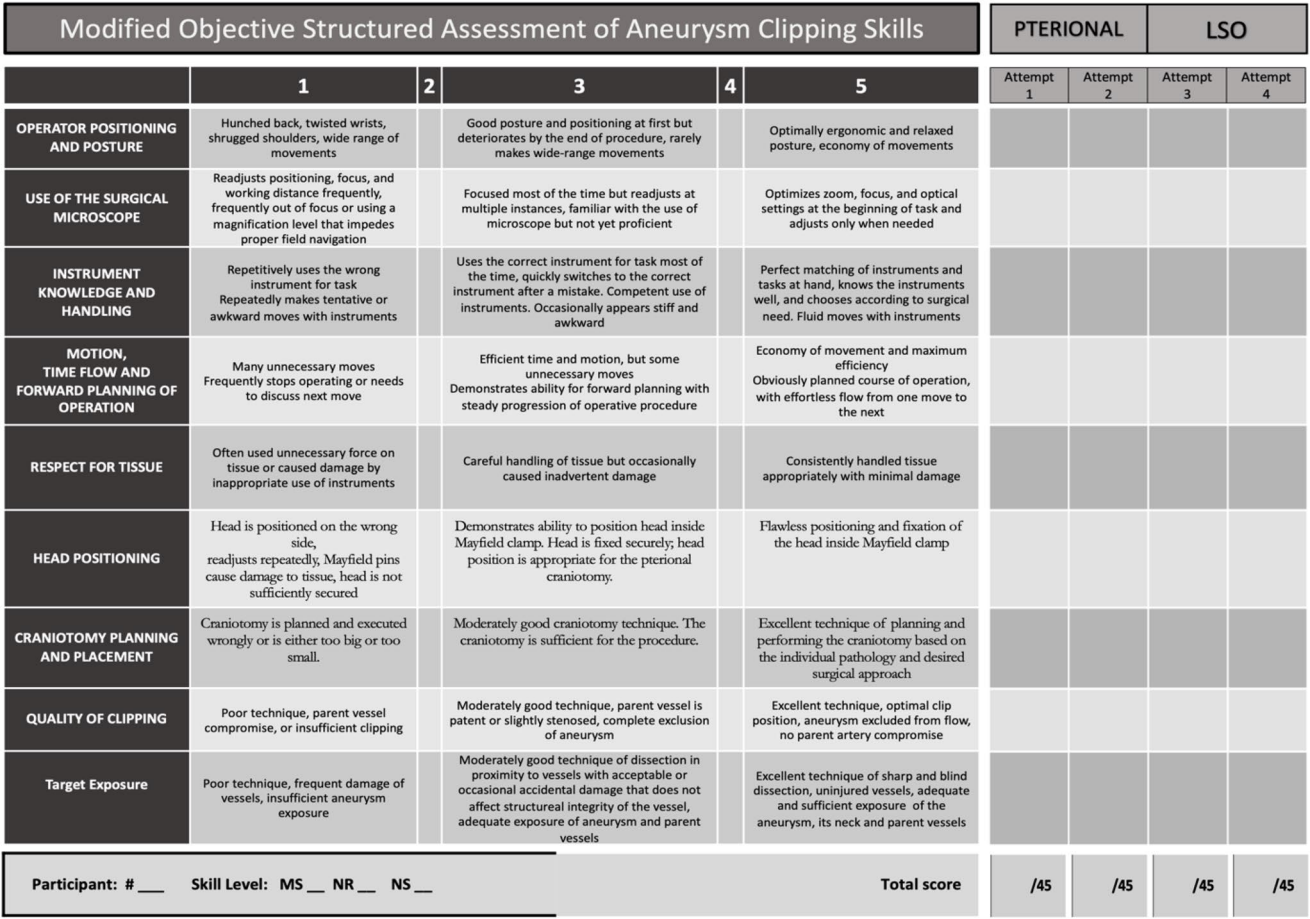
Modified objective structured assessment of aneurysm clipping skills (OSAACS)

## Results

### Subjective evaluation

**Fig. 9:**
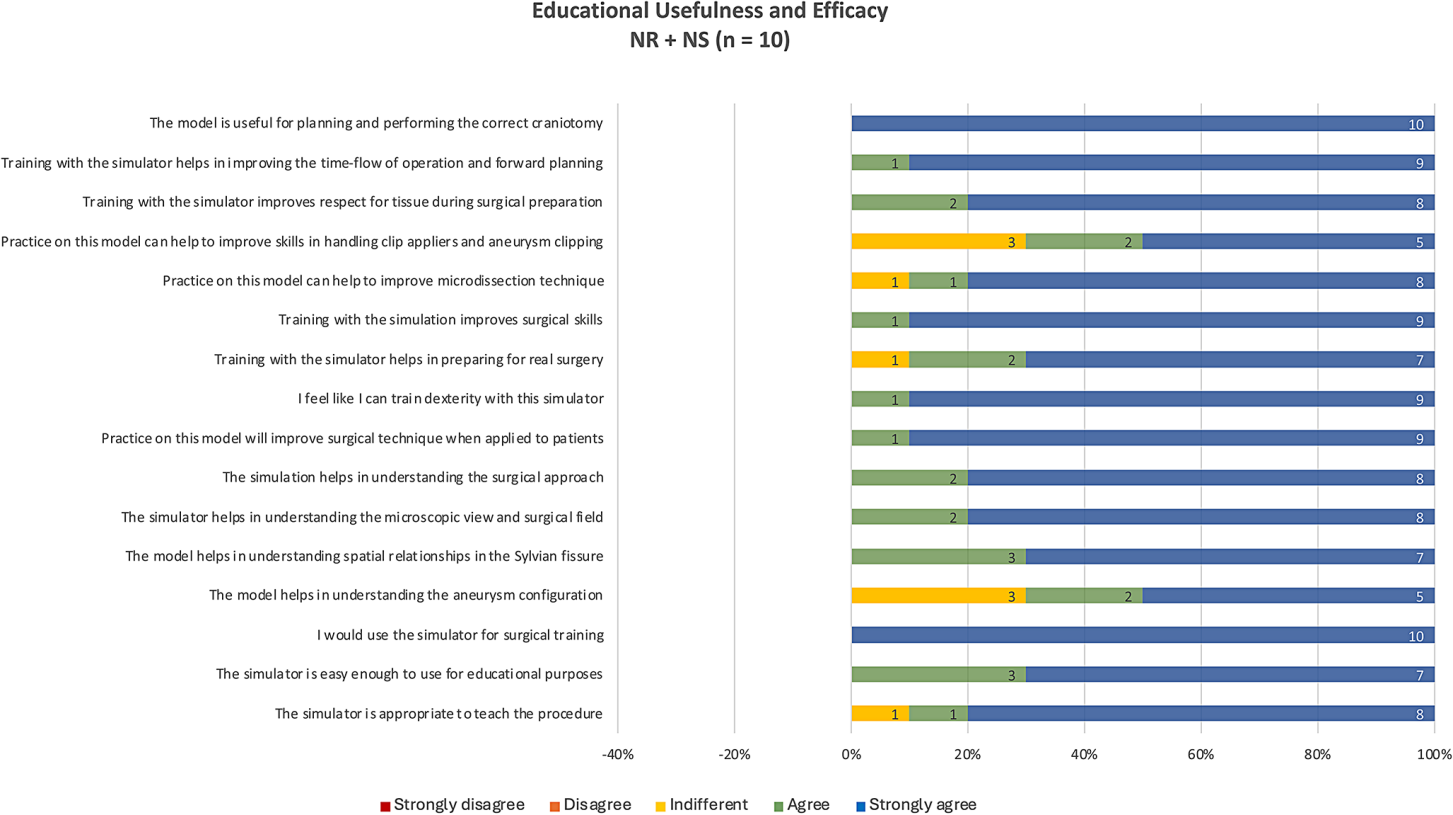
Average responses of participating residents and neurosurgeons (*n* = 10) on the simulator’s educational usefulness and efficacy derived from a 5-point Likert scale

**Fig. 10:**
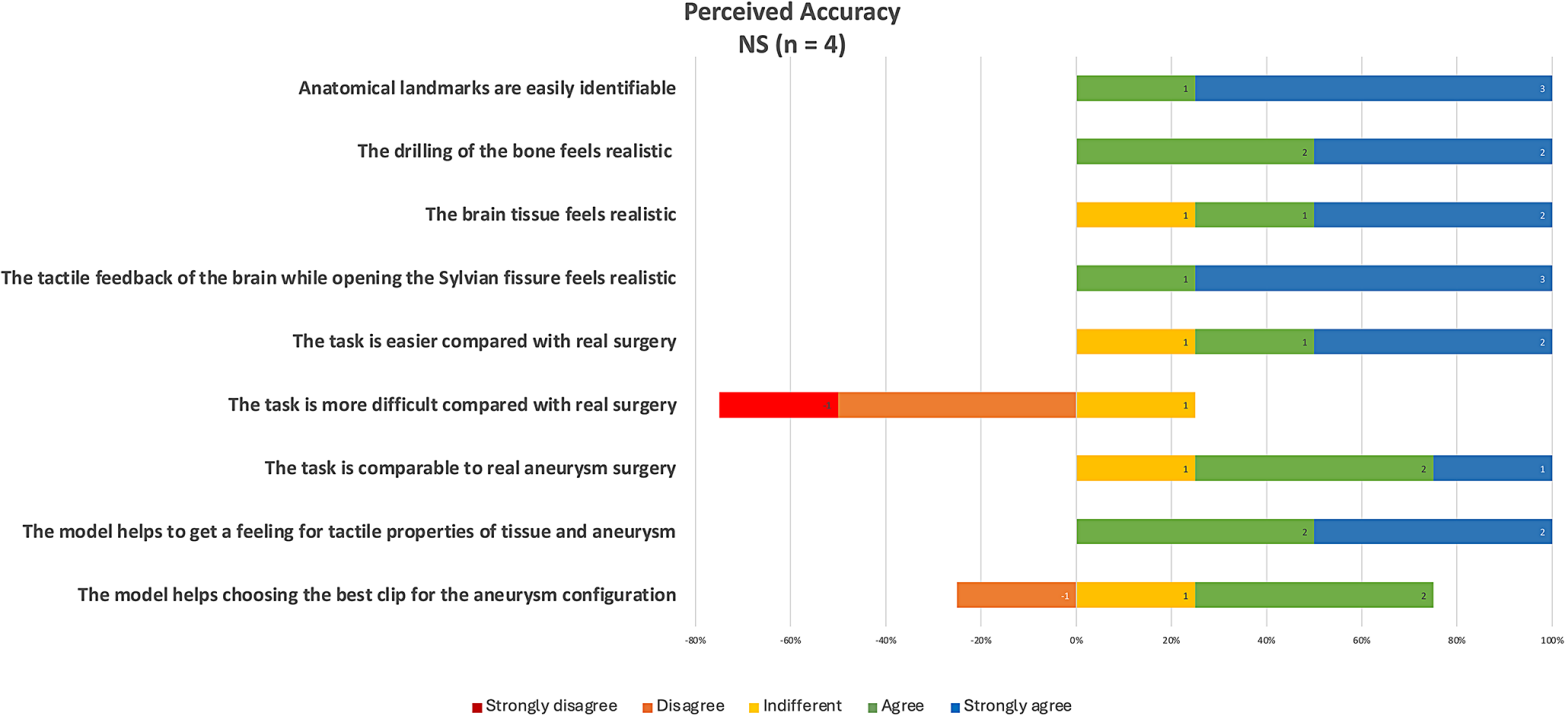
Average responses on realism and accuracy of the simulator as perceived by experienced neurosurgeons (*n* = 4) derived from a 5-point Likert scale

Face and content validities: the simulator was rated favorably by neurosurgical residents, and neurosurgeons with a mean score of 4.9 out of 5 regarding its educational usefulness in conveying and training key steps of the procedure and developing surgical skills. (Fig. 9) Experienced neurosurgeons perceived the simulator as highly accurate regarding tactile and anatomical realism (Fig. 10).

**Fig. 11:**
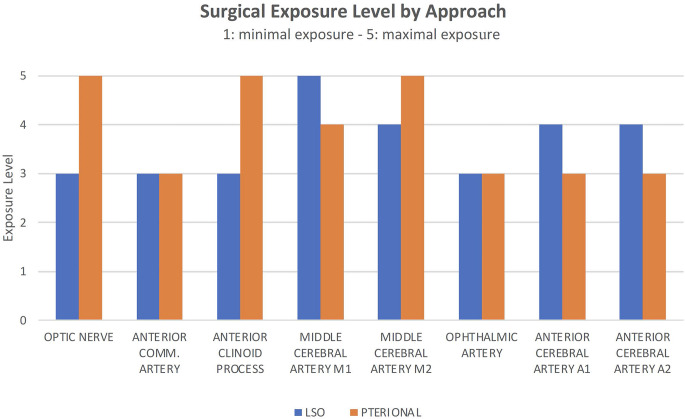
Comparative subjective grading of surgical exposure level of relevant anatomical structures via LSO and pterional approach as perceived by experienced neurosurgeons (*n* = 4)

The results of the subjective evaluation of the LSO and pterional approaches by experienced neurosurgeons regarding surgical exposure levels are shown in Fig. 11. In terms of accessibility, the LSO approach offered comparable exposure of the M1-segment and the bifurcation of the MCA as the pterional approach. This was particularly the case for MCA-bifurcation aneurysms with short M1-segments. However, while the LSO approach can provide access to the optic nerve and anterior clinoid process, its trajectory may limit exposure to structures that are more lateral or posterior without additional bone removal.

### Objective evaluation

**Fig. 12:**
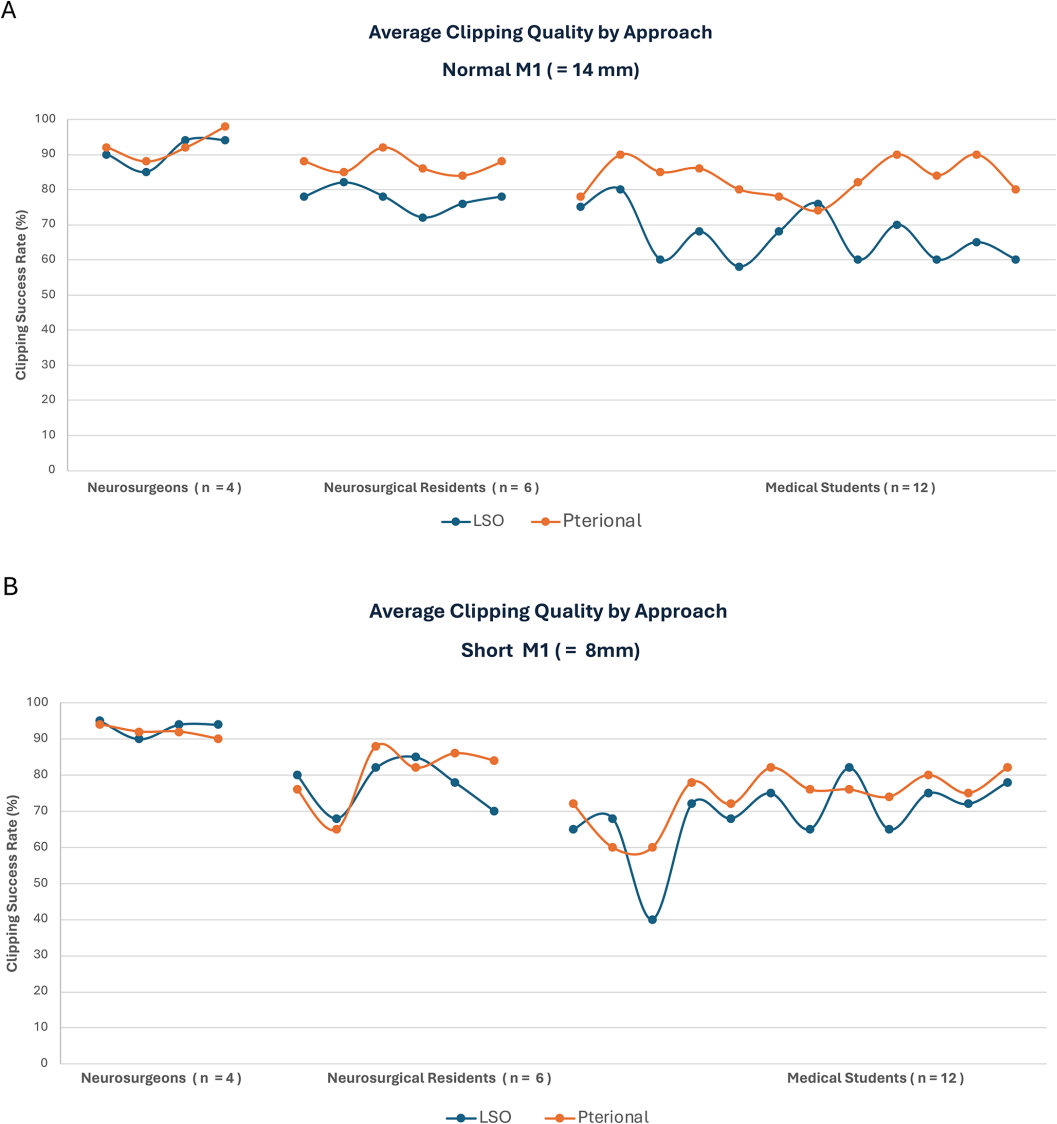
Average results in clipping quality by approach and group for MCA bifurcation-aneurysm with **(A)** an average length of the M1-segment (14 mm) and **(B)** a short M1-segment (8 mm)

Construct validity: the average results in clipping quality by approach and participant for MCA-bifurcation aneurysm with an average length of the M1-segment (14 mm) and a short M1-segment (8 mm) are presented in Fig. 12A and B. The placement of clips via the pterional approach was often easier for novice medical students and neurosurgical residents as it offerered superior ergonomics, resulting in higher clipping success rates, particularly for MCA -bifurcation aneurysms with an average M1-lenght.

**Fig. 13:**
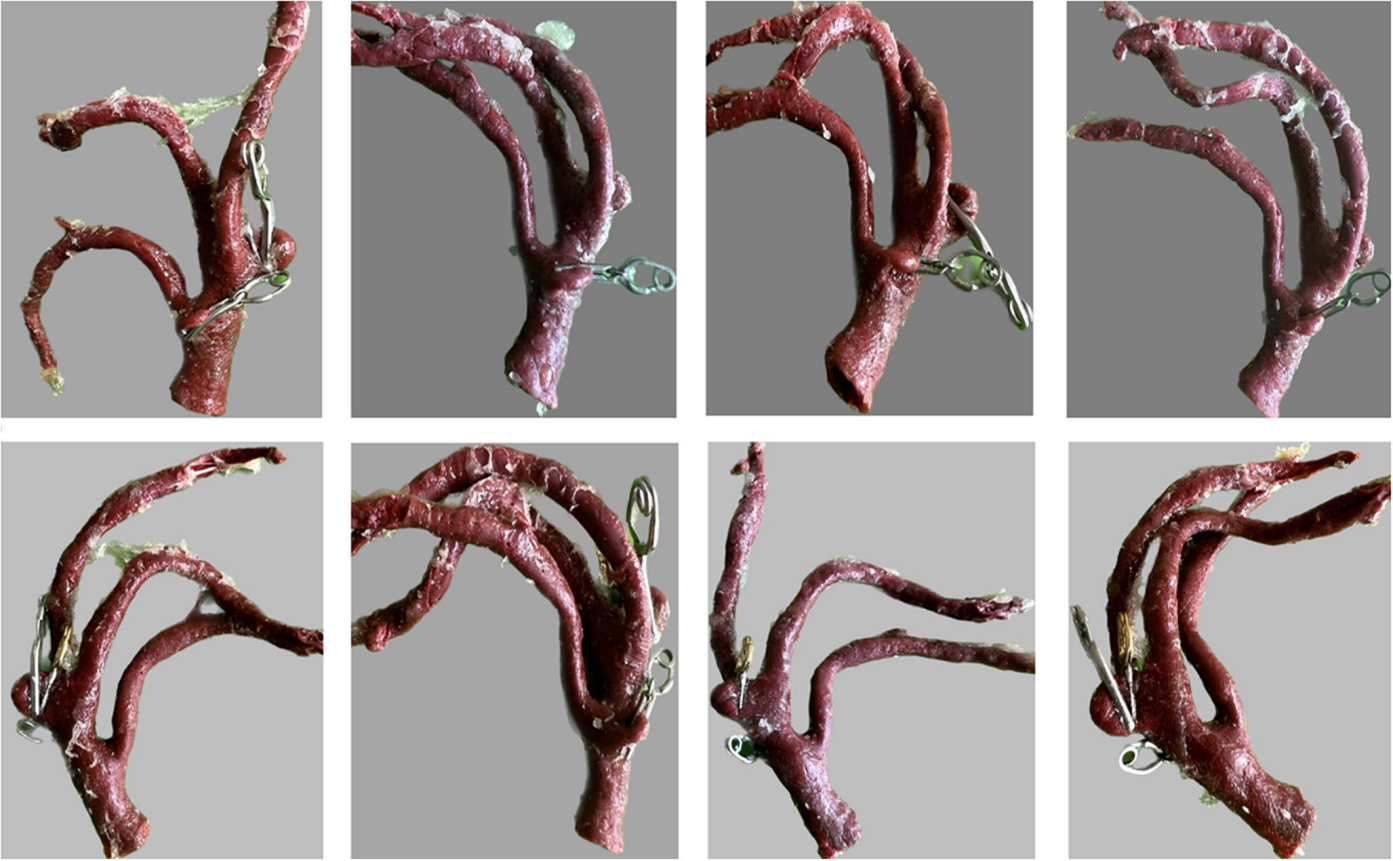
Post-simulation assessment of clipping quality of participating neurosurgeons based on OSAACS criteria revealing only a slight variation in clipping success between the two approaches

**Fig. 14:**
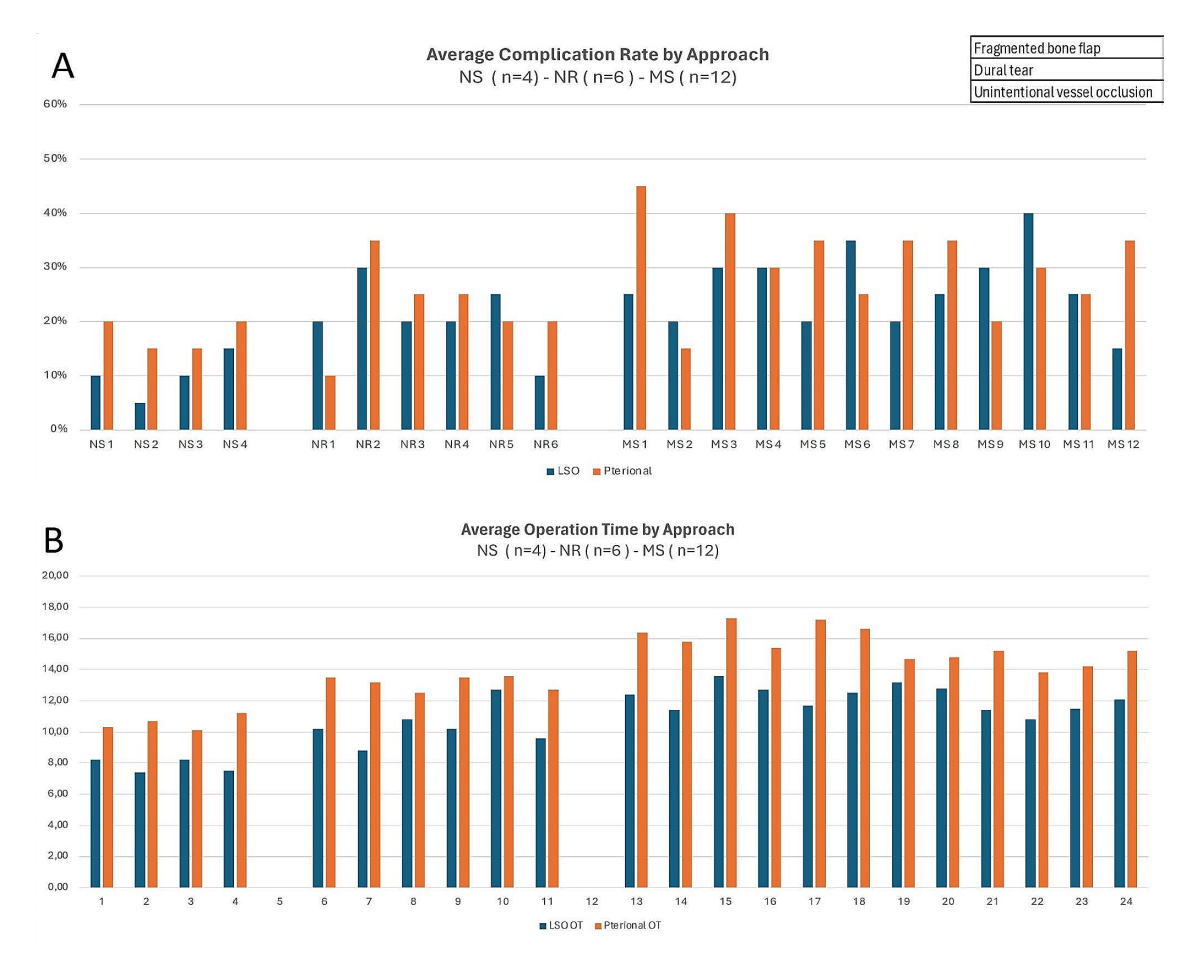
**(A)** shows the average complication rate by approach and group. Complications were divided in categories involving key steps of the procedure **(B)** provides an overview of the average procedure time by approach and group starting from head positioning to clipping of both aneurysms

However, among experienced neurosurgeons, the LSO approach offered comparable clipping success in MCA-bifurcation aneurysms with an average M1 length and even slightly higher clipping quality success in MCA variations with a short M1-segment, (Fig. 13) while, on average, offering lower complication rates and a shorter procedure time than the standard pterional approach. (Figs. 14A and B).

**Fig. 15:**
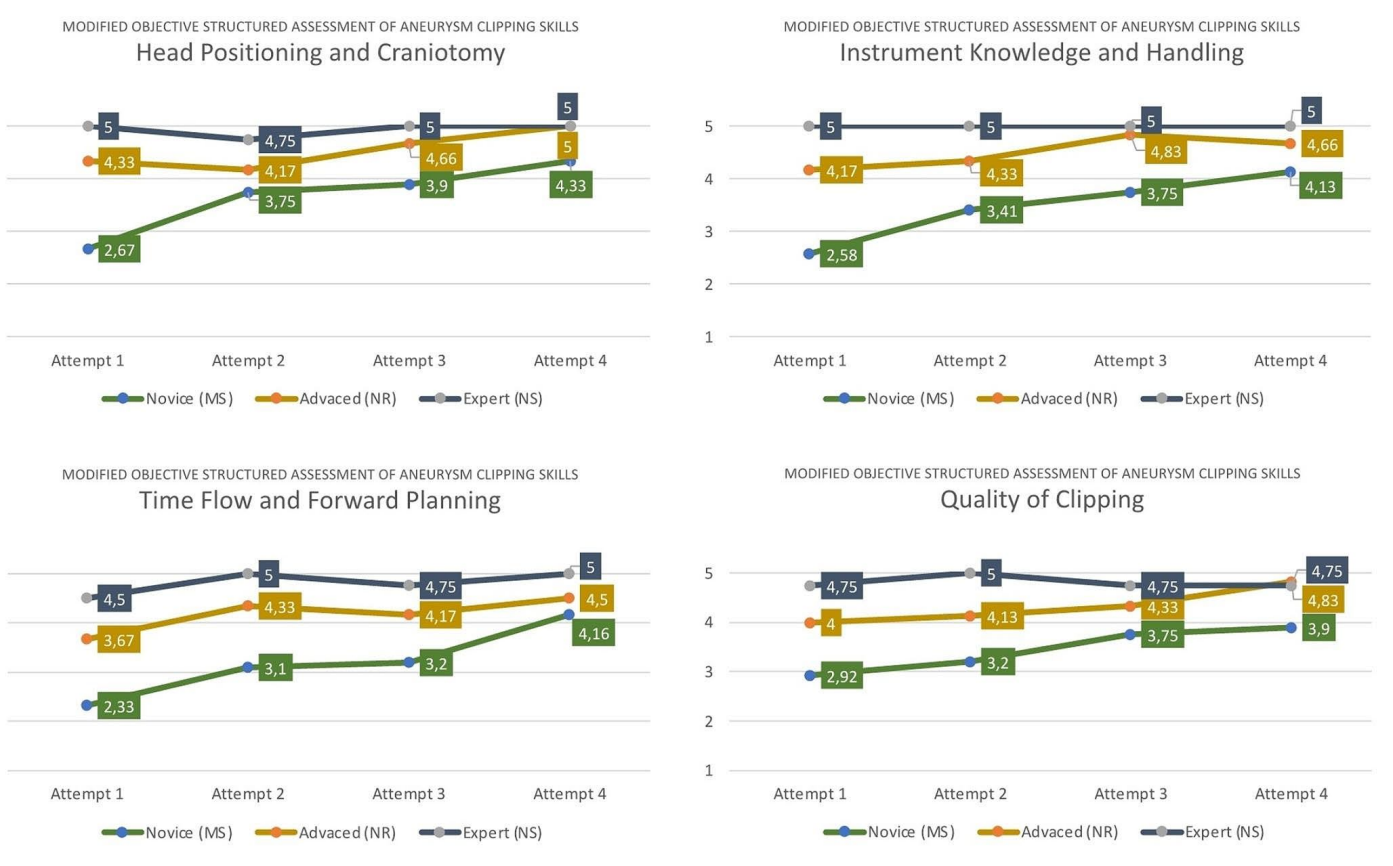
Objective assessment of performances of participating medicals students (MS), neurosurgical residents (NR) and neurosurgeons (NS) over time (attempts 1–4). Initial differences in surgical skills reflecting users’ abilities showcase a high construct validity of the model. Significant increase in surgical skills, particularly among novice students and resident neurosurgeons, demonstrate high efficacy of the Phantom.

The initial differences between the participating groups in clipping quality and other performance metrics based on OSAACS showcases a high construct validity of the simulator. Objective assessments of surgical clipping skills of the participating groups over a period of four attempts in four key metrics are presented in Fig. 15. The novice group experienced a rapid and considerable increase in accuracy, timing, and quality, underlining the efficacy of the simulator to convey microsurgical understanding and techniques.

Table 3 provides a comparative analysis of the LSO and pterional approaches based on subjective evaluations and objective assessments of the simulator and simulations.

**Table 3:** Comparative analysis of the pterional and lateral supraorbital approaches based on subjective evaluations and objective assessments of the simulator and performed simulations

Aspect	Pterional Approach	LSO Approach
Surgical Exposure	Comparable exposure for key structures, better for lateral/posterior structures	Comparable exposure for M1-segment and MCA bifurcation, better for shorter M1-segments
Ergonomics	Superior ergonomics	Good ergonomics but limited for more lateral/posterior structures
Operative Time	Longer	Shorter
Complication Rates	Higher	Lower among experts
Clipping Success	Higher success rates, especially for longer M1-segment aneurysms	Slightly higher success rates for shorter M1-segment aneurysms
Ease for Novices	Easier for novice medical students and residents	More time-efficient but technically challenging for novices

## Discussion

In this study, we investigated the utility of a state-of-the-art phantom model in juxtaposing the lateral supraorbital (LSO) and pterional approaches for treating unruptured middle cerebral artery (MCA) bifurcation aneurysms.

By facilitating a direct comparison between the two approaches, the presented model provided insights into the potential benefits of the LSO approach, such as comparable surgical exposure and clipping success with the potential for reduced surgical morbidity and improved postoperative recovery. The LSO approach, however, requires more technical expertise and precise surgical manipulation in a limited operative field. Traditionally, these skills are acquired through rigorous training and repetition. Simulation training has the potential to accelerate the learning curve by allowing surgeons to gain familiarity with these requirements and build confidence in performing the technique, thereby potentially improving patient outcomes.

The fundamental assumption of simulation-based training, however, is that skills acquired in simulated settings are transferable to the operative setting [22–24]. The more realistic the simulation, the more likely that it will help improve surgical skills such as dexterity and spatial awareness. At the same time, a lack of realism can lead to a negative learning effect and ultimately endanger patients’ lives.

The presented phantom model, designed to encompass MCA-bifurcation aneurysms at varying lengths of the M1-segment, allowed the participants to simulate the surgical experience closely, offering a safe and controlled environment for learning alternative approaches and skill refinement. The incorporation of accurate tactile and rheological properties enables trainees to develop a keen sense of touch, dexterity, and instrument handling that can be transferred to any neurosurgical procedures involving the use of microsurgical instruments and a microscope.

Limitations:

While the presented model demonstrates high content and construct validities, for the predictive validity of the simulator to be assessed, the simulator must be further improved to capture the complexity of real surgery and include specific microsurgical details like small perforators and venules, superficial veins, anatomical variability, and potential complications associated with them.

While the sequential improvement in skills observed among inexperienced participants indicates high efficacy, post-training performances in real-life scenarios would need to be evaluated to see if improvements on the simulator translate to real-world skill enhancements.

## Conclusion

The choice of surgical approach should be tailored to the individual patient and the specific characteristics of the aneurysm. The results of this study highlight the benefits of the LSO over the standard pterional approach such as reduced operation time and complication rates but also the challenges that a smaller approach entails. The results of the objective and subjective assessments of the presented model showcase the invaluable role of simulators in overcoming these challenges and, ultimately, advancing patient-specific treatment strategies. Future studies should continue to leverage phantoms simulators to explore and compare other surgical approaches, with the ultimate aim of accelerating the surgical learning curve and improving patient care.

## Data Availability

No datasets were generated or analysed during the current study.

## Declarations

Complete written informed consent was obtained from the patient for the publication of this study and accompanying images.

## References

[1] Wen HT, de Oliveira E, Tedeschi H, Andrade FC, Rhoton AL (2001) The pterional approach: Surgical anatomy, operative technique, and rationale. Operative Techniques Neurosurg 4(2):60–72. 10.1053/otns.2001.25567

[2] 1, Yasargil MG, Antic J, Laciga R, Jain KK, Hodosh RM, Smith RD (2005) Microsurgical pterional approach to aneurysms of the basilar bifurcation. Surg Neurol 63(6):491–499 951657

[3] Tra H, Huynh T, Nguyen B (2018) Minipterional and Supraorbital Keyhole craniotomies for ruptured anterior circulation aneurysms: experience at single Center. World Neurosurg 109:36–39. 10.1016/j.wneu.2017.09.058 28935549 10.1016/j.wneu.2017.09.058

[4] Lan Q, Zhang H, Zhu Q et al (2017) Keyhole Approach for clipping intracranial aneurysm: comparison of Supraorbital and Pterional Keyhole Approach. World Neurosurg 102:350–359. 10.1016/j.wneu.2017.02.025 28254535 10.1016/j.wneu.2017.02.025

[5] Lan Q, Zhu Q, Li G (2015) Microsurgical treatment of posterior cerebral circulation Aneurysms Via Keyhole approaches. World Neurosurg 84(6):1758–1764. 10.1016/j.wneu.2015.07.046 26211854 10.1016/j.wneu.2015.07.046

[6] Figueiredo EG, Deshmukh P, Nakaji P et al (2007) The minipterional craniotomy: technical description and anatomic assessment. Operative Neurosurg 61(5):256–265. 10.1227/01.neu.0000303978.11752.45 10.1227/01.neu.0000303978.11752.45 18091240

[7] Figueiredo EG, Welling LC, Preul MC et al (2016) Surgical experience of minipterional craniotomy with 102 ruptured and unruptured anterior circulation aneurysms. J Clin Neurosci 27:34–39. 10.1016/j.jocn.2015.07.032 26924181 10.1016/j.jocn.2015.07.032

[8] Hernesniemi J, Ishii K, Niemelä M et al (2005) Lateral supraorbital approach as an alternative to the classical pterional approach. In: Yonekawa Y, Keller E, Sakurai Y, Tsukahara T (eds) New trends of surgery for stroke and its Perioperative Management. Acta Neurochirurgica Supplements, vol 94. Springer-, pp 17–21. doi:10.1007/3-211-27911-3_4 10.1007/3-211-27911-3_4 16060236

[9] Choque-Velasquez J, Hernesniemi J (2018) One burr-hole craniotomy: lateral supraorbital approach in Helsinki Neurosurgery. Surg Neurol Int 9(1):156. 10.4103/sni.sni_185_18 30159200 10.4103/sni.sni_185_18 PMC6094498

[10] Cameron JL (1997) William Stewart Halsted. Our surgical heritage. Ann Surg 225(5):445–458. 10.1097/00000658-199705000-00002 9193173 10.1097/00000658-199705000-00002 PMC1190776

[11] Wright JR, Schachar NS (2020) Necessity is the mother of invention: William Stewart Halsted’s addiction and its influence on the development of residency training in North America. CJS 63(1):E13–E18. 10.1503/cjs.003319 10.1503/cjs.003319 PMC7828946 31944636

[12] Alaraj A, Luciano CJ, Bailey DP et al (2015) Virtual reality cerebral aneurysm clipping Simulation with Real-Time haptic feedback. Operative Neurosurg 11(1):52–58. 10.1227/NEU.0000000000000583 10.1227/NEU.0000000000000583 PMC4340784 25599200

[13] Rehder R, Abd-El-Barr M, Hooten K, Weinstock P, Madsen JR, Cohen AR (2016) The role of simulation in neurosurgery. Childs Nerv Syst 32(1):43–54. 10.1007/s00381-015-2923-z 26438547 10.1007/s00381-015-2923-z

[14] Oliveira LM, Figueiredo EG (2019) Simulation Training methods in neurological surgery. Asian J Neurosurg 14(2):364–370. 10.4103/ajns.AJNS_269_18 31143248 10.4103/ajns.AJNS_269_18 PMC6516032

[15] Akhtar KSN, Chen A, Standfield NJ, Gupte CM (2014) The role of simulation in developing surgical skills. Curr Rev Musculoskelet Med 7(2):155–160. 10.1007/s12178-014-9209-z 24740158 10.1007/s12178-014-9209-z PMC4092204

[16] Saalfeld S, Berg P, Neugebauer M, Preim B (2015) Reconstruction of 3d surface meshes for blood flow simulations of intracranial aneurysms. In Proceedings of the annual meeting of the german society of computer- and robot-assisted surgery (CURAC), pp. 163–168

[17] Amini A, Zeller Y, Stein KP et al (2022) Overcoming barriers in Neurosurgical Education: a Novel Approach to practical Ventriculostomy Simulation. Operative Neurosurg 23(3):225–234. 10.1227/ons.0000000000000272 10.1227/ons.0000000000000272 35972086

[18] Budday S, Sommer G, Haybaeck J, Steinmann P, Holzapfel GA, Kuhl E (2017) Rheological characterization of human brain tissue. Acta Biomater 60:315–329. 10.1016/j.actbio.2017.06.024 28658600 10.1016/j.actbio.2017.06.024

[19] Budday S, Ovaert TC, Holzapfel GA, Steinmann P, Kuhl E (2020) Fifty shades of brain: a review on the mechanical testing and modeling of Brain tissue. Arch Computat Methods Eng 27(4):1187–1230. 10.1007/s11831-019-09352-w

[20] Urvi S, Suman V, Subathra A (2023) Assessment of morphometric parameters of middle cerebral artery using CT angiography in a tertiary care hospital. Surg Radiol Anat 45(8):939–945. 10.1007/s00276-023-03148-1 37269412 10.1007/s00276-023-03148-1

[21] Belykh E, Miller EJ, Lei T et al (2017) Face, Content, and Construct Validity of an aneurysm clipping model using human placenta. World Neurosurg 105:952–960e2. 10.1016/j.wneu.2017.06.045 28647655 10.1016/j.wneu.2017.06.045

[22] Dawe SR, Pena GN, Windsor JA et al (2014) Systematic review of skills transfer after surgical simulation-based training. Br J Surg 101(9):1063–1076. 10.1002/bjs.9482 24827930 10.1002/bjs.9482

[23] Sturm LP, Windsor JA, Cosman PH, Cregan P, Hewett PJ, Maddern GJ (2008) A systematic review of skills transfer after Surgical Simulation Training. Ann Surg 248(2):166–179. 10.1097/SLA.0b013e318176bf24 18650625 10.1097/SLA.0b013e318176bf24

[24] Davids J, Manivannan S, Darzi A, Giannarou S, Ashrafian H, Marcus HJ (2020) Simulation for skills training in neurosurgery: a systematic review, meta-analysis, and analysis of progressive scholarly acceptance. Neurosurg Rev Published Online September 18. 10.1007/s10143-020-01378-0 10.1007/s10143-020-01378-0 PMC8338820 32944808

